# Patient reported symptoms and disabilities before and after neuroma surgery: a register-based study

**DOI:** 10.1038/s41598-023-44027-4

**Published:** 2023-10-11

**Authors:** Emma Dahlin, Malin Zimmerman, Erika Nyman

**Affiliations:** 1 Department of Biomedical and Clinical Sciences, Linköping University, Linköping, Sweden; 2 Varberg Hospital, Varberg, Sweden; 3 Department of Translational Medicine-Hand Surgery, Lund University, Malmö, Sweden; 4 Department of Orthopaedics, Helsingborg Hospital, Helsingborg, Sweden; 5 Department of Hand Surgery, Plastic Surgery, and Burns, Linköping University Hospital, Linköping, Sweden

**Keywords:** Trauma, Neuropathic pain

## Abstract

Residual problems may occur from neuroma despite surgery. In a 12-month follow-up study using national register data, symptoms, and disabilities related to surgical methods and sex were evaluated in patients surgically treated for a neuroma. Among 196 identified patients (55% men; lower age; preoperative response rate 20%), neurolysis for nerve tethering/scar formation was the most used surgical method (41%; more frequent in women) irrespective of affected nerve. Similar preoperative symptoms were seen in patients, where different surgical methods were performed. Pain on load was the dominating symptom preoperatively. Women scored higher preoperatively at pain on motion without load, weakness and QuickDASH. Pain on load and numbness/tingling in fingers transiently improved. The ability to perform daily activities was better after nerve repair/reconstruction/transposition than after neurolysis. Regression analysis, adjusted for age, sex, and affected nerve, showed no association between surgical method and pain on load, tingling/numbness in fingers, or ability to perform daily activities. Neuroma, despite surgery, causes residual problems, affecting daily life. Choice of surgical method is not strongly related to pre- or postoperative symptoms. Neurolysis has similar outcome as other surgical methods. Women have more preoperative symptoms and disabilities than men. Future research would benefit from a neuroma-specific ICD-code, leading to a more precise identification of patients.

## Introduction

A neuroma is considered the result of abnormal growth of regenerating nerve fibres, a process that may occur after a traumatic nerve injury. There are three different types of neuromas, which are defined by whether the connective tissue components of the nerve are intact or not, such as neuroma-in-continuity (proximal and distal nerve tissue components connected), end-neuroma (proximal and distal nerve ends separated)^1,2^ and the special case, where a nerve trunk is entrapped in a scar or is tethered to the surroundings (although, the injured nerve fibres do not always have an abnormal growth)^3^.

A neuroma, irrespective of type, can cause pain, paraesthesia, as well as sensory and motor loss^4–8^. Neuroma can induce severe disabilities with chronic pain^9^. Treatment of neuroma is complex and challenging, due to the complex mechanisms of chronic pain, why surgically identifying the anatomical origin may be more effective than medication^10^. Neuroma may be a source of chronic neuropathic pain, and therefore evaluating and developing surgical methods are highly current since the number of procedures is increasing^11^. The surgical procedures include active and passive methods, where active methods are used when the distal nerve end is available, and nerve function may recover after surgery. Passive methods are used in the absence of a distal nerve end, where a neuroma can be transposed into another surrounding^4,9^. When a nerve trunk is severely trapped in a scar or tethered to the surroundings, an exploration with neurolysis, with or without coverage with flaps, such as muscle or fat, may be indicated^3,12,13^.

Because of the shortage of detailed and well-defined data on patient characteristics and surgical methods for neuroma published in the literature, and also due to the complex symptomatology in patients with neuropathic pain, it is difficult to evaluate the outcome of various surgical methods. It is a challenge to evaluate outcomes based on medical records since there are no standardized methods for evaluation or any specific ICD-code present for neuroma, except for amputation neuroma^14^. Previous studies have mainly focused on postoperative outcome data^15,16^. During long-term follow-up, i.e., more than one year, symptoms, especially pain, improve after surgery, but remaining symptoms do occur^6,16^.

Therefore, there is a need for patient-reported outcome measures (PROMs) to evaluate the effects of surgical treatment of neuroma^17,18^. National quality registers may in these cases be useful to evaluate the treatment of neuroma with pre- and postoperative data^19,20^. Our aim was to perform a short-term follow-up on patients surgically treated for neuroma, using both active and passive surgical methods, including neurolysis, with data from a national quality register, with a specific focus on pre-and postoperative symptoms and disabilities related to surgical method and sex.

## Materials and methods

### Study design

Patients, with a suspected surgically treated neuroma in the upper limb between 2010 and 2021, were identified in the Swedish National Register for Hand Surgery HAKIR (hakir.se)^19^. The HAKIR register includes age, sex, time of surgery, ICD-10 codes and the NOMESCO Classification of Surgical Procedures (KVÅ code; https://klassifikationer.socialstyrelsen.se). There are no specific ICD-10 codes in the register that can specify the localization of the nerve injury/neuroma along the specific nerve, e.g., upper arm, elbow, forearm, or wrist. We could only distinguish the nerve injuries in those affecting a digital nerve or a major nerve trunk. No general or specific ICD-10 code(s) exists for neuroma, except for neuroma in amputation stump (T873). All possible ICD-10 were sorted out in the register, where a neuroma in continuity, an end-neuroma or a scarred or tethered nerve may have occurred and was surgically treated (T924, T873, D361, G563C, G568, S640, S641, S642, S643, S644, S540, S541, S542, S543). Initially, all applicable and relevant nerve injuries during the period were included for assessment. All possible patients were studied individually in the register. By combining the above-mentioned ICD diagnose codes with relevant surgical procedure(s) (KVÅ codes: ZZK00, ZZS10, ZZA50, QCE30, NDL29, NDQ29, NDH32, ZZR30, ZZQ10, ZZR05, QCE99, ACA11, ACC51, ACC21, ACB21, ACC52, ACA12, ACB22, ACC22, ACC42, ACC12, ACC43, ACB23, ACA13, ACC53, ACC23, ACB29, ACC49, ACA19, ACB19, ACC59, ACC29, ACC19) and affected nerve, the appropriate patients could be included with more accuracy.

**Figure 1:**
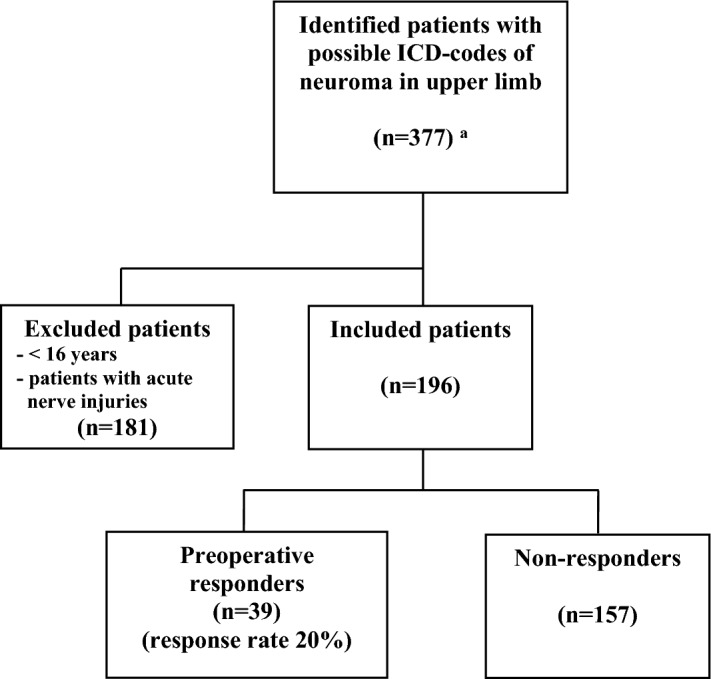
Flowchart illustrating identified patients that were diagnosed and surgically treated for a neuroma in the upper limb in the national registry for hand surgery (hakir.se) and evaluated for the study. ^a^For ICD codes and used surgical codes see “Materials and methods”.

Three persons (ED, MZ, and EN) went through the material together to choose the possible neuroma patients, who were treated for a single neuroma, and categorized the variables; nerve type, level of neuroma/initial nerve injury, and surgical method. In patients, where the diagnose code was T924 (sequel from injury of a nerve of the upper limb) and the surgical procedure was “nerve transposition of unspecific nerve” (ACC49) or “exploration of unspecific/other nerve” (ACA19), the type of nerve was categorized as digital nerve. Other variables in the register, such as reoperation (yes/no), or nerve injury (yes/no), were also used together with the diagnose and surgical codes to identify possible patients. If the ICD code started with S, indicating a nerve injury, the code for a re-operation in the register was required for inclusion. A flowchart is presented in Fig. 1 that explains the inclusion and exclusion of patients. In total, 196 patients (Fig. 1), with presumed neuroma diagnosis and surgical treatment, were found in the register and included in the study. The diagnosis “sequel from injury of nerve of upper limb” is in the present study defined and expressed as neuroma. Furthermore, the expression “neurolysis” is used to indicate surgery that also included “decompression” of a scarred or tethered nerve^3^.

### National register and questionnaires

HAKIR is a national quality register for hand surgery in Sweden, where almost all the departments of hand surgery are connected^19^, and patients with a age of 16 or older are included. Two questionnaires, HQ-8 and QuickDASH, are filled in by the patients preoperatively and at three and 12 months postoperatively.

The HAKIR-specific questionnaire HQ-8 includes eight questions based on the patient’s capability and symptoms in the affected hand, including questions about pain, stiffness, weakness, cold sensitivity, and their ability to work, sleep, and perform daily activities. No data were available from any PREM (patient-related evaluating measurement) questionaries or from any GROC (global rating of change) question. The responses are scored on a Likert scale between 0 and 100, where 0 is no problems and 100 is the worst case scenario^21^. The QuickDASH consists of eleven questions on a five-point Likert scale, where 1 is no difficulties and 5 is unable. In addition, if at least ten of eleven questions are answered a total QuickDASH score can be calculated, ranging from 0 to 100, where 100 represents the worst possible symptoms^22^. The Swedish version of the QuickDASH was used^22^.

### Statistical methods

Categorical qualitative variables and nominal data are presented as numbers (%). Chi-square or Fisher’s exact tests (if n < 5 in a group) were used to compare data between the categorical variables. The continuous data, scale variables, are presented as median [interquartile range; 25th–75th percentiles] and compared using the non-parametric Kruskal Wallis test, with Mann–Whitney U-test as a post-hoc test or as a single test. In conditions, where paired continuous values were analysed, the Friedman test and the Wilcoxon test were used. A linear regression analysis, adjusted for age, sex, and affected nerve (dichotomized as digital nerve and major nerve injury), was done to investigate any association between surgical method and pain on load, numbness/tingling in fingers, or ability to perform daily activities. Statistical significance was set at p < 0.05. Collected data was coded and analysed in IBM SPSS Statistics, version no 28 (Armonk, USA).

### Ethics

All patients provide written informed consent before inclusion in the register. They are informed that the data only will be used to develop and ensure the quality of care, compile statistics, and conduct healthcare-related research and is thus only used by, or disclosed to, those using the data for any of these three purposes. All reporting is done with anonymized data. The study was approved by the Swedish Ethical Review Authority (permission number 2021-06232-02). All methods were performed in accordance with relevant guidelines and regulations, including the Declaration of Helsinki.

## Results

### Patient population

The basic characteristics of the 196 included patients surgically treated for a neuroma are presented in Table 1. Among these, 88/196 (45%) were women and 108/196 (55%) were men. Half of the included patients were treated for a neuroma located on a digital nerve (n = 98, 50%). The distribution among the major nerve trunks in the upper limb was almost equal. The most common surgical method was neurolysis of a tethered or scarred nerve (81/196; 41%), with no difference regarding the affected nerve (Tables 1 and 2). Nerve repair with suture or nerve reconstruction (58/196; 30%) and nerve transposition (42/196; 21%) were also performed as well as covering with soft tissue (15/196, 8%). Women were older than men (p < 0.001), but with an equal distribution regarding the type of affected nerve and location of neuroma (Table 1). Considering the surgical method, neurolysis of a tethered or scarred nerve was more common among women, whereas nerve repair with suture/nerve reconstruction as well as nerve transpositions were more common in men (p = 0.003; Tables 1 and 2). Characteristics between the patients treated with the active surgical method nerve repair/nerve reconstruction and nerve transposition, a passive method, did not differ regarding age, sex, type of nerve, or location of neuroma (Supplementary Table S1, also, too few patients to evaluate the outcome of surgery).

**Table 1:** Basic characteristics of all included patients surgically treated for neuroma in the upper limb.

	Total population (n = 196)	Women (n = 88; 45%)	Men (n = 108; 55%)	P-value
Age (years)	45 [32–57]	52 [41–61]	40 [26–51]	** < 0.001**
Type of nerve ^a^				
Median nerve	30 (15)	13 (15)	17 (16)	0.91
Radial nerve	27 (14)	13 (15)	14 (13)	
Ulnar nerve	21 (11)	11 (13)	10 (9)	
Digital nerve	98 (50)	44 (50)	54 (50)	
Location of neuroma or initial nerve injury ^b^				
Hand/wrist	123 (63)	57 (65)	66 (61)	0.32
Forearm	26 (13)	14 (16)	12 (11)	
Surgical method				
Neurolysis	81 (41)	48 (55)	33 (31)	0.003*
Nerve repair with suture/reconstruction ^c^	58 (30)	19 (22)	39 (36)	
Nerve transposition ^d^	42 (21)	13 (15)	29 (27)	
Covering ^e^	15 (8)	8 (9)	7 (7)	

Patients were selected from the national hand surgical register (hakir.se) 2010–2021.

Values are median [IQR; 25th–75th percentiles] or n (%). Significant p-values (p <0.05) are marked in bold. P-values based on Mann–Whitney U-test (continuous data) or Chi-squared test (or Fisher´s exact test if n < 5 in a group; nominal data, independent samples; sex).

* In cases, where surgical method showed significant difference, further test with Chi-squared test was done to see where any difference was seen. Significant differences were seen between neurolysis and nerve repair with sutures/reconstruction (p = 0.002) and between neurolysis and nerve transposition (p = 0.002).

^a ^Data missing in 20 patients; 7 among women (8%) and 13 among men (12%).

^b ^Data missing in 47 patients; 17 among women (19%) and 30 among men (28%).

^c ^Excision of neuroma and nerve repair with suture, repair with conduit or nerve reconstruction with auto- or allograft.

^d ^Nerve transposition with and without conduit.

^e ^Surgical flaps or full skin transplantation, including muscles or fat, or excision of surgical scar.

**Table 2:** Comparison between surgically treated patients with neuroma in the upper limb divided by neurolysis and nerve repair with suture/nerve reconstruction or nerve transposition.

	Neurolysis (n = 81)	Nerve repair with sutures/nerve reconstruction or nerve transposition (n = 100)	P-value
Age (years)	45 [35–59]	44 [31–55]	0.20
Sex (women/men)	48 (59)/33 (41)	32 (32)/68 (68)	**<0.001**
Type of nerve ^a^			
Median nerve	19 (24)	10 (10)	0.09
Radial nerve	10 (12)	16 (16)	
Ulnar nerve	7 (9)	14 (14)	
Digital nerve	44 (54)	54 (54)	
Location of neuroma or initial nerve injury ^b^			
Hand/wrist	50 (62)	65 (83)	0.68
Forearm	12 (15)	13 (17)	

Values are median [IQR; 25th–75th percentiles] or n (%).

P-values based on Mann–Whitney U-test (continuous data) or Chi-squared test (or Fisher´s exact test if n < 5 in a group, nominal data, independent samples; sex).

Significant p-values (p <0.05) are marked in bold.

^a ^Data missing in 1 patient in neurolysis group and 6 patients in nerve repair with sutures/nerve reconstruction/nerve transposition group.

^b ^Data missing in 19 patients in neurolysis group and 22 patients in nerve repair with sutures/nerve reconstruction/nerve transposition group.

Among the 196 patients, 55 of the patients responded to the two questionnaires at least at one of the time points. No significant difference was observed between responders and non-responders concerning age, sex, type of injured nerve, and surgical method, although location of neuroma was more frequently observed in the forearm among non-responders (p = 0.015; Supplementary Table S2). Among the preoperative responders, there was an equal distribution among women and men, with no significant differences concerning age, type of nerve, location of neuroma or nerve injury, or surgical methods between sexes (Table 3).

**Table 3:** Basic characteristics of all preoperative responding patients surgically treated for a neuroma in upper limb.

	Responders preoperative (n = 39)	Women (n = 19; 49%)	Men (n = 20; 51%)	P-value
Age (years)	47 [33–61]	54 [41–61]	40 [26–60]	0.08
Type of nerve ^a^				
Median nerve	6 (15)	3 (16)	3 (15)	0.72
Radial nerve	1 (3)	0 (0)	1 (5)	
Ulnar nerve	3 (8)	2 (11)	1 (5)	
Digital nerve	26 (67)	13 (68)	13 (65)	
Location of neuroma or initial nerve injury ^b^				
Hand/wrist	27 (69)	13 (68)	14 (70)	0.48
Forearm	2 (5)	2 (11)	0 (0)	
Surgical method				
Neurolysis	22 (56)	13 (68)	9 (45)	0.08
Nerve repair with suture/reconstruction ^c^	8 (21)	3 (16)	5 (25)	
Nerve transposition ^d^	7 (18)	1 (5)	6 (30)	
Covering ^e^	2 (5)	2 (11)	0 (0)	

Values are median [IQR; 25th–75th percentiles] or n (%).

P-values based on Mann–Whitney U-test (continuous data) or Chi-squared test (or Fisher´s exact test if n < 5 in a group; nominal data; independent samples; sex).

Significant p-values (p <0.05) are marked in bold.

^a ^Data missing in 3 patients; 1 patient among women and 2 among men.

^b ^Data missing in 10 patients; 4 patients among women and 6 among men.

^c ^Excision of neuroma and nerve repair with suture, repair with conduit or nerve reconstruction with auto- or allograft.

^d ^Nerve transposition with and without conduit.

^e ^Surgical flaps and full skin transplantation, including muscles or fat, or excision of surgical scar.

### The HQ-8 and QuickDASH questionnaires

Of the total population of 196 persons, 39 patients responded to the preoperative questionnaires, giving a response rate of 20%. Preoperatively, women scored higher than men in the HQ-8 questionnaire regarding pain on motion without load (p = 0.045), weakness (p = 0.003), and total QuickDASH score (p = 0.039; Table 4). No other differences were observed. There were no statistical differences concerning the preoperative symptoms and disabilities based on HQ-8 and QuickDASH between the patients surgically treated with neurolysis and nerve repair/reconstruction/transposition (Supplementary Table S3), despite a larger proportion of women (Table 2).

**Table 4:** Preoperative response of HQ-8 questions and total QuickDASH score for patients surgically treated for a neuroma in the upper limb divided by sex.

	Preoperative responders (n = 39)	Women (n = 19)	Men (n = 20)	P-value
Pain on load	70 [40–85]	80 [50–85]	55 [40–88]	0.48
Pain on motion without load	30 [10–70]	60 [30–70]	23 [2–41]	**0.045**
Pain at rest	30 [3–60]	40 [20–60]	10 [2–32]	0.09
Stiffness ^a^	50 [16–70]	60 [38–73]	33 [10–68]	0.10
Weakness ^b^	50 [28–80]	75 [45–81]	33 [13–53]	**0.003**
Numbness/tingling in fingers ^c^	55 [30–55]	55 [25–82]	55 [33–90]	0.39
Cold sensitivity ^d^	59 [22–80]	40 [0–90]	60 [48–80]	0.40
Ability to perform daily activities	60 [40–73]	60 [50–80]	45 [22–70]	0.12
Total QuickDASH score ^e^	50 [27–66]	59 [41–75]	43 [20–52]	**0.039**

HQ-8 questions are an abbreviation of HAKIR Questionnaire 8. QuickDASH stands for the short version of disabilities of the arm, shoulder and hand questionnaire. HQ-8 questions and total QuickDASH score evaluated preoperatively. 0 is no problems and 100 is worst case scenario.

Values are median [IQR; 25th–75th percentiles].

P-values based on Mann–Whitney U-test (continuous data). Significant p-values (p <0.05) are marked in bold.

HQ-8 = Hand Surgery Questionnaire used in national quality register from Sweden.

^a ^Data missing in 1 woman.

^b ^Data missing in 1 woman.

^c ^Data missing in 1 woman.

^d ^Data missing in 1 man.

^e ^Data missing in 1 woman.

**Figure 2:**
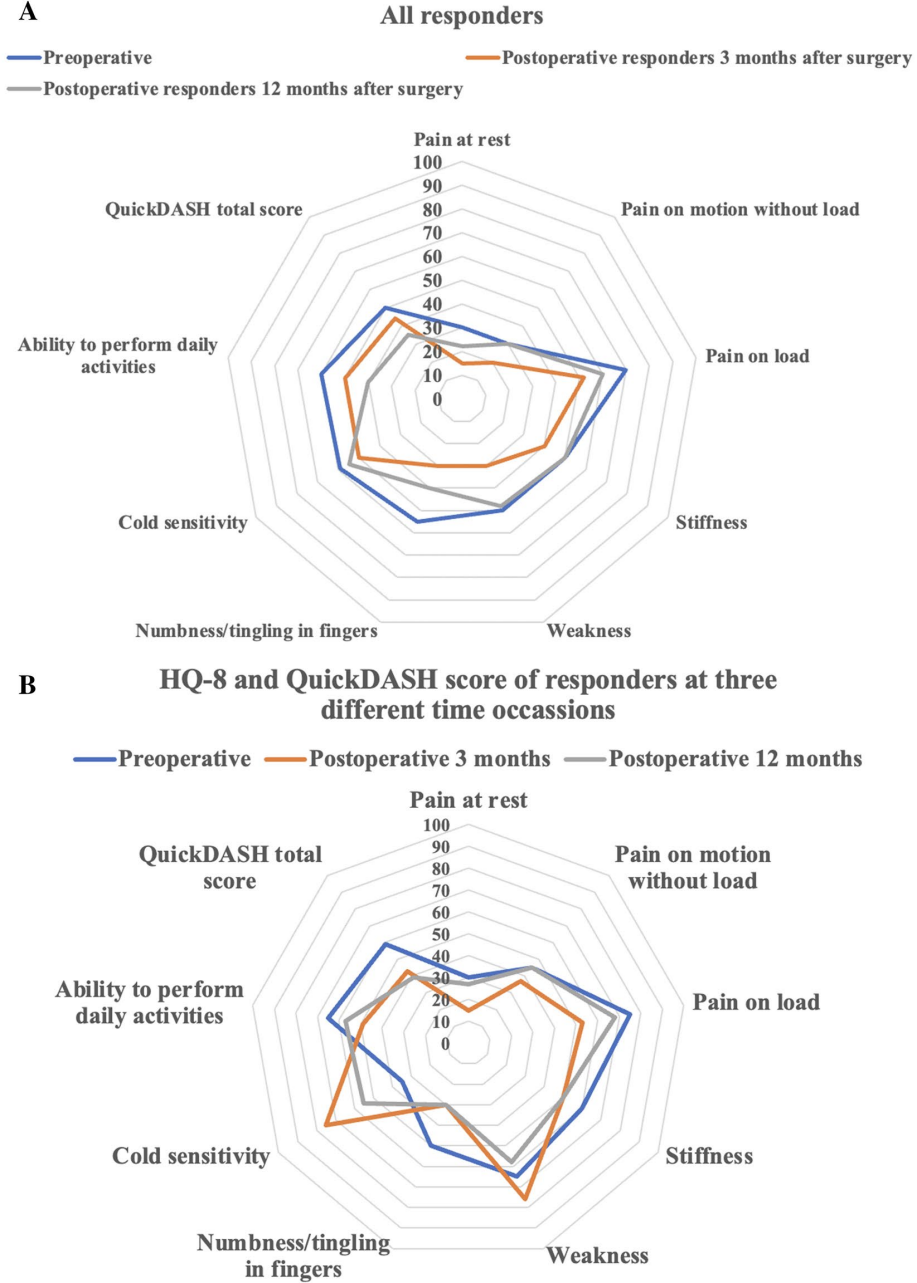
Spider diagrams showing the outcome of neuroma surgery among (**a**) all responders and (**b**) eight patients responding at all three time points. Data are based on the HQ-8 questionnaire consisting of eight specific questions and the total score of the QuickDASH questionnaire. HQ-8 questions are an abbreviation of HAKIR Questionnaire 8. QuickDASH stands for the shortened disabilities of the arm, shoulder and hand questionnaire. HQ-8 questions and total QuickDASH score evaluated preoperatively. 0 is no problems and 100 is worst case scenario.

Table 5 and Fig. 2a present the results of the HQ-8 and total QuickDASH scores among the responding patients at all three different time points, including all patients. There was no significant difference between the different time points, except for that numbness/tingling in fingers was scored higher preoperatively than at three months postoperatively (p = 0.037). Furthermore, eight patients responded to the questionnaires on all three occasions, which is illustrated in Table 6 and Fig. 2b. There were some trends in the development of symptoms over time, with some improvement or worsening at three months after surgery. However, only pain on load showed a significant difference (p = 0.028) between the preoperative response and the response at three months postoperative; likewise, the three-month response differed from the 12-month response. Thus, the score was reduced at three months, indicating an amelioration in symptomatology, to later (12 months) being increased again concerning pain on load (Table 6). The results at three and 12 months were also pooled and compared to preoperative data, concerning HQ-8 and total QuickDASH score in a separate analysis. The results showed an improvement in numbness/tingling in fingers postoperatively (Supplementary Table S4), in accordance with non-pooled data (Table 5).

**Table 5:** Pre- and postoperative response of HQ-8 questions and total QuickDASH score for patients surgically treated for a neuroma in upper limb.

	Preoperative (n = 39)	3 months postoperative (n = 20)	12 months postoperative (n = 24)	P-value
Age (years)	47 [33–61]	53 [38–61]	51 [38–61]	0.81
Pain on load	70 [40–85]	52 [23–70]	60 [40–80]	0.17
Pain on motion without load	30 [10–70]	20 [12–59]	30 [14–60]	0.86
Pain at rest	30 [3–60]	15 [6–65]	22 [1–50]	0.79
Stiffness	50 [16–70]	40 [20–70]	50 [23–70]	0.79
Weakness	50 [28–80]	30 [19–80]	48 [20–70]	0.93
Numbness/tingling in fingers ^a^	55 [30–55]	30 [10–60]	40 [13–69]	0.037 *
Cold sensitivity ^b^	59 [22–80]	50 [10–80]	55 [20–55]	0.10
Ability to perform daily activities	60 [40–73]	50 [30–70]	40 [10–60]	0.13
Total QuickDASH score ^c^	50 [27–66]	44 [18–66]	35 [22–70]	0.55

HQ-8 questions are an abbreviation of HAKIR Questionnaire 8. QuickDASH stands for the short version of disabilities of the arm, shoulder and hand questionnaire. HQ-8 questions and total QuickDASH score evaluated preoperatively and postoperatively at three and 12 months. 0 is no problems and 100 is worst case scenario.

Values are median [IQR; 25th–75th percentiles].

P-values based on Kruskal–Wallis test and when significant further test with Mann–Whitney U-test. Significant p-values (p <0.05) are marked in bold.

* Significant differences were seen between preoperative responders and postoperative responders after three months, but not compared to 12 months concerning numbness/tingling in fingers.

^a ^Data missing in 1 patient in 3 months postoperative group.

^b ^Data missing in 1 patient in 3 months postoperative group.

^c ^Data missing in 2 patients in 3 months postoperative group.

**Table 6:** Response of HQ-8 questions and total QuickDASH score pre- and postoperatively (three and 12 months) for eight patients surgically treated for a neuroma in upper limb responding at all three time points.

	Preoperative (n= 8)	3 months postoperative (n = 8)	12 months postoperative (n = 8)	P-value
Pain on load	75 [50–89]	53 [33–75]	68 [53–78]	0.028 *
Pain on motion without load	45 [33–68]	37 [20–70]	45 [30–60]	0.63
Pain at rest	30 [23–68]	15 [10–70]	27 [20–65]	0.32
Stiffness	60 [40–64]	50 [10–68]	50 [23–68]	0.55
Weakness	65 [38–83]	76 [33–80]	58 [40–70]	0.23
Numbness/tingling in fingers ^a^	50 [30–88]	30 [10–80]	30 [13–74]	0.58
Cold sensitivity	35 [0–85]	75 [23–80]	55 [8–88]	0.77
Ability to perform daily activities	65 [18–84]	49 [33–76]	57 [43–78]	0.66
QuickDASH score ^b^	59 [43–77]	43 [18–73]	39 [28–65]	0.57

HQ-8 questions are an abbreviation of HAKIR Questionnaire 8. QuickDASH stands for the short version of disabilities of the arm, shoulder and hand questionnaire. HQ-8 questions and total QuickDASH score evaluated preoperatively and postoperatively at three and 12 months (only eight individuals responding at all three time points). 0 is no problems and 100 is worst case scenario.

Values are median [IQR; 25th–75th percentiles].

P-values based on Friedman test (paired continuous data) and when significant further test with Wilcoxon test. Significant p-values (p < 0.05)are marked in bold.

^* ^Significant differences were seen between preoperative responders and postoperative responders after three months, and between postoperative responders after three months (p = 0.019) and postoperative responders after 12 months (p = 0.03) concerning pain on load.

^a^ Data missing in 1 patient in the 3 months postoperative group.

^b^ Data missing in 1 patient in the 3 months postoperative group.

The postoperative comparison between the surgical methods neurolysis and nerve repair/reconstruction/transposition showed only a statistical difference in the ability to perform daily activities, where the patients after neurolysis had higher scores (Supplementary Table S5). Further linear regression analysis showed no association between the surgical methods (neurolysis being reference against nerve repair/reconstruction/transposition) adjusted for age, sex and affected nerve concerning pain on load (unstandardized beta − 6, 95% CI – 30 to 18; p = 0.62), numbness/tingling in fingers (unstandardized beta − 18, 95% CI − 42 to 6; p = 0.14) or ability to perform daily activities (unstandardized beta − 18, 95% CI − 43 to 7; p = 0.14). In the adjusted analysis for numbness/tingling in fingers, where the variable affected nerve was dichotomized into digital (reference) and major nerve injury, an association with numbness/tingling in fingers and a major nerve injury was found (unstandardized beta 41, 95% CI 14–68; p = 0.005).

## Discussion

The present study shows that patients with a neuroma, preoperatively evaluated from a national register, greatly suffer from symptoms and disabilities, particularly concerning pain on load, cold sensitivity, and impaired ability to perform daily activities. This is indicated also by a high total QuickDASH score, where women experienced more symptoms and disabilities preoperatively than men. Preoperative pain on load presented the highest score of all HQ-8 symptoms, which confirms the complexity of neuropathic pain and is probably a leading indication for surgery.

The preoperative total QuickDASH score was similar to another study on surgically treated neuroma, also based on evaluation with QuickDASH, showing a preoperative score of 50 compared to postoperative score of 35 and 40^23^. In agreement, surgery among the patients in our study had no great impact on the symptoms and disabilities with only a small and possibly transient improvement in pain on load and numbness/tingling in fingers during the 12-month follow-up. Interestingly, the evaluated symptoms and disabilities, using the same questionnaires as in the present study, may essentially remain in 114 patients surgically treated for neuroma at a follow-up study with a median of 51 months postoperatively, except that pain at rest and pain on motion without load may show some improvement over time^24^.

The described preoperative symptoms that the present patients with neuroma experience, such as pain, stiffness, weakness, numbness/tingling, and cold sensitivity, harm their daily lives. Such symptoms reduce the functionality of the hand, which decreases the work capacity and in the long run also leads to high unemployment^23^. Our population was quite young and in their working years. It is possible that sequels, such as neuroma formation following an injury, could affect work capacity and lead to sick leave. Another study on primary surgically treated digital nerve injuries showed a score of 20 on pain on load (HQ-8 questionnaire) in contrast to the present score of 60, indicating severe problems and a different complexity with neuroma^25^. On the other hand, surgically treated major nerve injuries show a higher score on pain on load, approaching 40, which is more in accordance with the present study^26^. The total postoperative QuickDASH score at 12 months after primary repair of a digital (score 29) or a major nerve injury (score 17–34) is more in line with our study^25,26^. The regression analysis did not reveal any association with the type of surgical method and pain on load, numbness/tingling in fingers, or ability to perform daily activities, but a major nerve injury was associated with numbness/tingling in fingers. This indicates that a neuroma in a major nerve may have more sensory symptoms, but was not associated with more pain on load or disabilities to perform daily activities. This indicates the complexity of symptoms and disabilities in surgically treated neuroma patients.

In the present study, the preoperative responders had the highest score on pain on load together with the ability to perform daily activities, which confirms the difficulties with the execution of their daily occupations and activities when having pain, where employment status, duration of pain, CRPS symptoms and smoking are prognostic factors for the outcome of surgery^23^. Interestingly, the patients undergoing nerve repair/reconstruction/transposition had a better postoperative score at 12 months concerning their ability to perform daily activities than the ones that were treated with neurolysis, despite a similar distribution of affected nerve and preoperative symptoms and disabilities. However, no association could be found in the linear regression analysis when adjusted for age, sex, and affected nerve regarding ability to perform daily activities.

A neuroma, which is superficially located in its anatomical location, such as a neuroma in a digital nerve in a finger, a radial sensory nerve branch, or the median nerve at the wrist level, may cause more symptoms and disabilities compared to a neuroma in a more deeply located nerve trunk. Thus, at these locations, no existing tissue is thick enough to provide with a cushion effect thereby protecting the repaired nerve or a neuroma. In contrast to superficially located nerves, the deeply located nerves are more protected by the surroundings, for example the median nerve at mid forearm level that is covered by muscle tissue, and therefore are not as frequently injured as the superficially located ones. The anatomical localization is therefore decisive for the risk of injury and residual problems, irrespective of surgery. However, one should also consider any concomitant injuries to other structures in the traumatized area. In this study, we do not have any information about surgical procedures in detail at the primary event.

In the present study,  women were older and reported more preoperative symptoms and disabilities, with higher total QuickDASH score, which is similar to other hand surgical conditions, such as osteoarthritis of the first CMC joint and carpal tunnel syndrome^27,28^. Neurolysis was the most frequently used surgical method in the present study and dominated among women, indicating that neuroma can also be related to a scarred or tethered nerve^6^. Indeed, the present definition of neuroma includes tethering and scar formation around the nerve by a widespread and previously presented definition^3^, where neurolysis is a relevant surgical method (20% of studies in the meta-analysis included neurolysis and coverage)^14^. Despite that neurolysis was the most frequently used technique in the present study (41%), being significantly more common among women, the affected nerve did not differ, indicating that the procedures were rather equally done among digital nerves and the major nerve trunks. In the stratified sub-analysis in the large meta-analysis by Poppler et al., 91% of the patients with neuroma and > 24 months duration of pain improved by neurolysis and covering with healthy soft tissue, which was significantly better than excision of neuroma and repair (30%)^14^. Interestingly, no differences were observed when the duration of symptoms was < 24 months. The present data indicate that the preoperative symptoms and disability were similar between the patients treated with neurolysis and nerve repair/reconstruction/transposition, which may indicate that the indication for surgery was similar for the two surgical groups. Data from a national register study can utilize the individual patient's preoperative symptoms and disabilities in contrast to retrospective studies^6,15,24,29^.

Patients undergoing surgery with neurolysis had a higher score in the ability to perform daily activities, but no association was found in the regression analysis when adjusted for age, sex, and affected nerve. Nevertheless, treatment of neuroma related to tethering and scar formation around the nerve are important aspects, including the history and duration of pain, in creating algorithms for diagnosis and surgical treatment of neuroma. Elliot et al. emphasized that the different types of neuroma may have different pain modalities, concluding that the surgical method should not only be related to the anatomical position of the neuroma but also to the pain symptomatology^3^, which is partly in contrast to the present data indicating similar preoperative symptoms and disabilities. We consider neurolysis an important surgical method as tethering and scar formation around the nerve can cause neuromas.

In the separate analysis of a fewer number of patients that replied at all three time points, only pain on load showed a temporary improvement. The initial improvement, followed by deterioration, of the score of pain on load is interesting and understandable from a clinical perspective in the surgery of patients with neuroma. In addition, there was also a transient improvement in numbness/tingling in fingers, which may also be understandable from a clinical perspective. Numbness/tingling in fingers seemed to be associated with affection of the major nerves compared to the digital nerves, which is clinically understandable. No other scores were significantly changed by surgery, although trends of variations could be seen among the other symptoms. Again, this illustrates the complexity of neuroma treatment, where all previously prognostic and involved factors may influence the outcome of surgery^14,23^. We did not have access to further details about the present patients. Nevertheless, neuroma patients may show a reduction in pain after surgery, indicated by a significant decrease in VAS (visual analogue scale), an improvement in quality of life^23^ and a reduction in the presence of pain judged by the surgeon^6^.

The present population had a slight majority of men and a median age of 45 years, which is similar to our recent study^6^ and a published metanalysis^14^. However, the patient characteristics sex, and age are different from recent studies on repaired and reconstructed digital and major nerve injuries, based on national registers, where a majority were men at a lower median age^25,26^. This can be interpreted as that the present group of patients with neuroma may represent a general population with nerve injuries, where late residual problems may have developed resulting in an indication for surgery. Procedures, such as nerve repair with suture or reconstruction and nerve transpositions, were more common in men, which is noticeable since digital nerves were most frequently affected. A recent study, based only on digital nerve injuries, showed that active methods, such as repair/reconstruction, had a better outcome compared to excision and implantation (i.e., transposition)^15^; the latter, however, being a less common procedure in the present patients. Our data did not allow any comparison between nerve repair/reconstruction and nerve transposition.

Surgery after nerve injuries, such as digital nerves, should not be delayed since it may have a severe impact on the patient’s quality of life^8^, impaired sensory function^30^, and with a risk of postoperative neuropathic pain^31^. In addition, pure motor nerve injuries may not cause residual problems, such as pain, or paraesthesia/tingling, but remaining problems, despite surgery, are often related to weakness in the affected hand and arm^32,33^. An improperly repaired or reconstructed motor nerve can cause muscle dysfunctions and secondary pain problems, such as cramps in the arm and hand due to compensation. Thereby, an injury or a neuroma in a motor nerve may cause residual problems interpreted by the affected individual as pain on load. However, generally, a surgically treated motor nerve, with nerve repair or nerve reconstruction, might have a better ability to regain function than a sensory nerve^34^. Instead of directly suturing injured nerves, there are alternative methods for nerve reconstruction, such as nerve conduits, autologous nerve grafting, processed nerve allografts, and nerve transfers^35^.

In addition, surgery for untreated nerve injuries may reduce the medication of opioids and non-opioids^10^. In the present study, we had no data on pre- or postoperative medication, such as opioids and gabapentinoid drugs, but this may be a focus in future studies using combinations of national registers. Active methods, being the second most frequently used methods in our study, focus on restoration of function, which may include more extensive and complex procedures, but are probably more crucial in treating pain^4^. Passive methods, such as nerve transposition, may also work well for pain relief, which was not possible to analyse in the present study. An individual approach to each treated patient with a symptomatic neuroma is important, where all the characteristics of the problems are carefully judged. Analysis of specific biomarkers and genes may be helpful, utilizing the concepts of precision medicine to better identify neuroma patients that are suitable for medical or surgical treatment^36–40^.

Patient-related outcome measures before and after surgery are well needed in further investigations to develop an evaluation of the outcome of different types of neuroma surgery, where large populations must be investigated. This is indicated by the present study using data from a smaller population with a risk of underpowering. The preoperative response rate was 20% and the postoperative 10% (three months) and 12% (12 months). However, data from other national registers^28^ and follow-up studies^8^ also show a low response rate. Even with a low response rate, the results do not need to be less valid according to a previously published article^41^, but one should consider the possibility that the response rate may be influenced by the patient's education and income^42^. One may also argue concerning the low response rate in two ways; the responders may be patients who are not pleased or pleased with the results. The present limited success of the surgery may indicate that the patients generally are unpleased with surgery and that the outcome should be interpreted with caution. The results from other studies have shown that a lower response rate is normally seen in follow-up studies, where men tend to respond less often than women which diverges from the present study^43^. In addition, there is a need to use PROMs with specific questions concerning pain modalities^18,44^ with the intention of future development of the procedures in neuroma surgery. A strength of the present study is the defined follow-up time (rather precisely three and 12 months), which differs from other published articles^6,24,29,45^.

## Conclusion

We conclude that patients with neuroma formation following a nerve injury, despite neuroma surgery, greatly suffer from symptoms and disabilities affectingt daily activities. The choice of surgical method is not related to pre- or postoperative symptoms. Neurolysis, frequently used in women, and used for neuroma with tethering/scar formation around nerves, has a similar surgical outcome to the other presently evaluated surgical procedures. Women are older and have more severe preoperative problems than men. Neuroma surgery should focus on pain relief, but our results indicate only a temporary improvement in pain on load and numbness/tingling in fingers at short-time follow-up. The most important point is the prevention of neuroma formation, which is achieved by a proper diagnosis and adequate surgical procedures at the primary event. Future research would benefit from the creation of a neuroma-specific ICD code, leading to a more precise identification of patients.

### Supplementary Information

Supplementary Tables.

## Data Availability

Public access to the present data is restricted by the Swedish Authorities (Public Access to Information and Secrecy Act), but data from national quality registers can be made available for researchers after a special review that includes approval of a research project by both the national Swedish Ethical Review Authority and the authorities’ data safety committees.

## References

[1] Regal S, Tang P (2019). Surgical management of neuromas of the hand and wrist. J. Am. Acad. Orthop. Surg..

[2] Bolleboom A, Boer K, de Ruiter GCW (2021). Clinical outcome for surgical treatment of traumatic neuroma with a processed nerve allograft: Results of a small prospective case series. J. Foot Ankle Surg..

[3] Elliot D, Sierakowski A (2011). The surgical management of painful nerves of the upper limb: A unit perspective. J. Hand. Surg. Eur..

[4] Eberlin KR, Ducic I (2018). Surgical algorithm for neuroma management: A changing treatment paradigm. Plast. Reconstr. Surg. Glob. Open.

[5] Lans J (2022). Long-term opioid use following surgery for symptomatic neuroma. J. Reconstr. Microsurg..

[6] Nyman E, Dahlin E, Gudinge H, Dahlin LB (2022). Surgically treated neuroma in upper extremity: Patient characteristics and factors influencing outcome of surgery. Plast. Reconstr. Surg. Glob. Open.

[7] Stonner MM, Mackinnon SE, Kaskutas V (2021). Predictors of functional outcome after peripheral nerve injury and compression. J. Hand Ther..

[8] Felder JM, Ducic I (2021). Chronic nerve injuries and delays in surgical treatment negatively impact patient-reported quality of life. Plast. Reconstr. Surg. Glob. Open.

[9] Sayegh A, Jaloux C, Witters M, Mayoly A, Kachouh N (2023). Update on upper limb neuroma management. J. Craniofac. Surg..

[10] Felder JM, Ducic I (2021). Impact of nerve surgery on opioid and medication use in patients with chronic nerve injuries. Plast. Reconstr. Surg. Glob. Open.

[11] Drinane JJ, Ruter D, Eberlin KR, Bialowas C (2021). A longitudinal assessment of the surgical treatment of symptomatic neuromas and their surgical management in the American College of Surgeons National Surgical Quality Improvement Program database. J. Plast. Reconstr. Aesthet. Surg..

[12] Dahlin LB, Lekholm C, Kardum P, Holmberg J (2002). Coverage of the median nerve with free and pedicled flaps for the treatment of recurrent severe carpal tunnel syndrome. Scand. J. Plast. Reconstr. Surg. Hand Surg..

[13] Dahlin LB, Salö M, Thomsen N, Stütz N (2010). Carpal tunnel syndrome and treatment of recurrent symptoms. Scand. J. Plast. Reconstr. Surg. Hand Surg..

[14] Poppler LH (2018). Surgical interventions for the treatment of painful neuroma: A comparative meta-analysis. Pain.

[15] Lans J (2020). Patient-reported outcomes following surgical treatment of symptomatic digital neuromas. Plast. Reconstr. Surg..

[16] Gottlieb RW, Westenberg RF, Chen NC, Coert JH, Eberlin KR (2021). Long-term outcomes after surgical treatment of radial sensory nerve neuromas: Patient-reported outcomes and rate of secondary surgery. Plast. Reconstr. Surg..

[17] Domeshek LF (2017). Surgical treatment of neuromas improves patient-reported pain, depression, and quality of life. Plast. Reconstr. Surg..

[18] Wang Y, Sunitha M, Chung KC (2013). How to measure outcomes of peripheral nerve surgery. Hand Clin..

[19] Arner M (2016). Developing a national quality registry for hand surgery: Challenges and opportunities. EFORT Open Rev..

[20] Zimmerman M (2019). Open carpal tunnel release and diabetes: A retrospective study using PROMs and national quality registries. BMJ Open.

[21] Carlsson IK, Ekstrand E, Åström M, Stihl K, Arner M (2021). Construct validity, floor and ceiling effects, data completeness and magnitude of change for the eight-item HAKIR questionnaire: A patient-reported outcome in the Swedish National Healthcare Quality Registry for hand surgery. Hand Therapy.

[22] Gummesson C, Ward MM, Atroshi I (2006). The shortened disabilities of the arm, shoulder and hand questionnaire (QuickDASH): validity and reliability based on responses within the full-length DASH. BMC Musculoskelet. Disord..

[23] Stokvis A, van der Avoort DJC, van Neck JW, Hovius SER, Coert JH (2010). Surgical management of neuroma pain: A prospective follow-up study. Pain.

[24] Dahlin E, Gudinge H, Nyman E, Dahlin LB (2023). Neuromas cause severe residual problems at long-term despite surgery. Sci. Rep..

[25] Frostadottir D (2022). Cold sensitivity, functional disability and predicting factors after a repaired digital nerve injury. Sci. Rep..

[26] Frostadottir D, Ekman L, Zimmerman M, Dahlin LB (2022). Cold sensitivity and its association to functional disability following a major nerve trunk injury in the upper extremity-A national registry-based study. PLoS One.

[27] Linde F, Rydberg M, Zimmerman M (2022). Surgically treated carpal tunnel syndrome and ulnar nerve entrapment at the elbow in different occupations and their effect on surgical outcome. J. Occup. Environ. Med..

[28] Wilcke M, Roginski M, Åström M, Arner M (2020). A registry based analysis of the patient reported outcome after surgery for trapeziometacarpal joint osteoarthritis. BMC Musculoskelet. Disord..

[29] Guse DM, Moran SL (2013). Outcomes of the surgical treatment of peripheral neuromas of the hand and forearm: a 25-year comparative outcome study. Ann. Plast. Surg..

[30] Kim JS (2018). A Systematic Review of Prognostic Factors for Sensory Recovery After Digital Nerve Reconstruction. Ann Plast Surg.

[31] de Lange JWD (2022). Prevalence of post-traumatic neuropathic pain after digital nerve repair and finger amputation. J. Plast. Reconstr. Aesthet. Surg..

[32] He B (2014). Factors predicting sensory and motor recovery after the repair of upper limb peripheral nerve injuries. Neural Regen. Res..

[33] Menorca RM, Fussell TS, Elfar JC (2013). Nerve physiology: Mechanisms of injury and recovery. Hand Clin..

[34] Chemnitz A, Björkman A, Dahlin LB, Rosén B (2013). Functional outcome thirty years after median and ulnar nerve repair in childhood and adolescence. J. Bone Joint Surg. Am..

[35] Yi C, Dahlin LB (2010). Impaired nerve regeneration and Schwann cell activation after repair with tension. Neuroreport.

[36] Zimmerman M, Nilsson P, Dahlin LB (2023). Exposure to hand-held vibrating tools and biomarkers of nerve injury in plasma: A population-based, observational study. BMJ Open.

[37] Ising E (2021). Quantitative proteomic analysis of human peripheral nerves from subjects with type 2 diabetes. Diabet. Med..

[38] Oki G (2012). Metallothionein deficiency in the injured peripheral nerves of complex regional pain syndrome as revealed by proteomics. Pain.

[39] Toia F, Giesen T, Giovanoli P, Calcagni M (2015). A systematic review of animal models for experimental neuroma. J. Plast. Reconstr. Aesthet. Surg..

[40] Ising E (2023). Quantification of heat shock proteins in the posterior interosseous nerve among subjects with type 1 and type 2 diabetes compared to healthy controls. Front. Neurosci..

[41] Stirling PHC (2022). Nonresponder bias in hand surgery: Analysis of 1945 cases lost to follow-up over a 6-year period. J. Hand Surg. Eur..

[42] Finsen V (2015). The influence of education and income on responses to the QuickDASH questionnaire. J. Hand Surg. Eur..

[43] Westenberg RF (2020). What factors are associated with response rates for long-term follow-up questionnaire studies in hand surgery?. Clin. Orthop. Relat. Res..

[44] Mathieson S, Maher CG, Terwee CB, Folly de Campos T, Lin CW (2015). Neuropathic pain screening questionnaires have limited measurement properties: A systematic review. J. Clin. Epidemiol..

[45] El-Gammal YT, Cardenas-Mateus L, Tsai TM (2023). Outcomes of surgical treatment of peripheral neuromas of the hand and forearm. J. Brachial Plex. Peripher. Nerve Inj..

