# Supplementary material for: Patient reported symptoms and disabilities before and after neuroma surgery: a register-based study
Source: Sci Rep. 2023 Oct 11;13:17226. doi: 10.1038/s41598-023-44027-4 (PMC10567846; doi:10.1038/s41598-023-44027-4)
Supplement: Supplementary file 1 — Supplementary Tables. [file 41598_2023_44027_MOESM1_ESM.docx]

**Supplementary Table S1.** Comparison between surgically treated patients with neuroma in the upper limb divided by nerve repair with suture/nerve reconstruction and nerve transposition.

|  | **Nerve repair with sutures/nerve reconstruction (n=58)** | **Nerve transposition**  **(n=42)** | **P-value** |
| --- | --- | --- | --- |
| **Age (years)** | 43 [42-52] | 44 [27-57] | 0.51 |
| **Sex (women/men)** | 19 (33) / 39 (76) | 13 (31) / 29 (69) | 0.56 |
| **Type of nerve ^a^** |  |  | 0.12 |
| Median nerve | 9 (16) | 1 (2) |  |
| Radial nerve | 7 (12) | 9 (21) |  |
| Ulnar nerve | 9 (16) | 5 (12) |  |
| Digital nerve | 30 (52) | 24 (57) |  |
| **Location of neuroma or initial nerve injury ^b^** |  |  | 0.12 |
| Hand/wrist | 41 (79) | 24 (57) |  |
| Forearm | 11 (19) | 2 (5) |  |

Values are median [IQR; 25^th^ – 75^th^ percentiles] or n (%). P-values based on Mann-Whitney U-test or Chi-squared test (or Fisher´s exact test if n < 5 in a group; independent samples; sex). Statistically significant if p<0.05; marked as bold.

*^a^ Data missing in 3 patients in nerve repair with suture/nerve reconstruction group and 3 missing patients in nerve transposition group.*

*^b^ Data missing in 6 patients in nerve repair with suture/nerve reconstruction group and 16 missing patients in nerve transposition group.*

**Supplementary Table S2.** Total population and subgroups of surgically treated patients with neuroma in the upper limb divided by responders (at any time occasions) and non-responders.

|  | **Total population**  **(n=196)** | **Responders**  **(n=55)** | **Non-responders**  **(n=141)** | **P-value** |
| --- | --- | --- | --- | --- |
| **Age (years)** | 45 [32-57] | 45 [32-61] | 44 [32-55] | 0.54 |
| **Sex (women/men)** | 88(45)/108(55) | 26 (47)/29(53) | 64(43)/85(57) | 0.40 |
| **Type of nerve ^a^** |  |  |  | 0.17 |
| Median nerve | 30 (15) | 7 (13) | 23 (16) |  |
| Radial nerve | 27 (14) | 4 (7) | 23 (16) |  |
| Ulnar nerve | 21 (11) | 4 (7) | 17 (12) |  |
| Digital nerve | 98 (50) | 33 (60) | 65 (46) |  |
| **Location of neuroma or initial nerve injury ^b^** |  |  |  | **0.015** |
| Hand/wrist | 123 (63) | 36 (65) | 87 (62) |  |
| Forearm | 26 (13) | 2 (4) | 24 (17) |  |
| **Surgical method** |  |  |  | 0.18 |
| Neurolysis | 81 (41) | 27 (49) | 54 (38) |  |
| Nerve repair with suture/reconstruction ^c^ | 58 (30) | 10 (18) | 48 (34) |  |
| Nerve transposition ^d^ | 42 (21) | 13 (24) | 29 (21) |  |
| Covering ^e^ | 15 (8) | 5 (9) | 10 (7) |  |

Values are median [IQR; 25^th^ – 75^th^ percentiles] or n (%). P-values based on Mann-Whitney U-test and Chi-2 test. Statistically significant if p<0.05; marked as bold.

^a^ *Data missing in 20 patients; 7 among responders, 13 among non-responders.*

*^b^ Data missing in 47 patients; 17 among responders and 30 among non-responders.*

*^c^ Excision of neuroma and nerve repair with suture, repair with conduit or nerve reconstruction with auto- or allograft.*

*^d^ Nerve transposition with and without conduit.*

*^e^ Surgical flaps and full skin transplantation, including muscles or fat, or excision of surgical scar.*

**Supplementary Table S3.** Preoperative response of HQ-8 questions and total QuickDASH score in patients surgically treated for neuroma in the upper limb.

|  | **Neurolysis**  **(n=22)** | **Nerve repair with sutures/nerve reconstruction or**  **nerve transposition (n=15)** | **P-value** |
| --- | --- | --- | --- |
| **Age (years)** | 46 [34-61] | 44 [31-55] | 0.95 |
| **Sex (women/men)** | 13/9 (59/41) | 4/11 (27/73) | 0.05 |
| **Pain on load** | 80 [48-90] | 50 [40-80] | 0.41 |
| **Pain on motion without load** | 35 [8-73] | 30 [10-70] | 0.76 |
| **Pain at rest** | 30 [10-70] | 30 [2-50] | 0.37 |
| **Stiffness** | 60 [8-70] | 40 [18-61] | 0.59 |
| **Weakness ^a^** | 50 [25-80] | 54 [20-80] | 0.77 |
| **Numbness/tingling in fingers** | 50 [10-90] | 66 [30-100] | 0.26 |
| **Cold Sensitivity ^b^** | 50 [20-90] | 70 [28-80] | 0.72 |
| **Ability to perform daily activities** | 60 [40-80] | 40 [20-73] | 0.34 |
| **Total QuickDASH score ^c^** | 53 [43-76] | 41 [16-59] | 0.08 |

HQ-8 questions are an abbreviation of HAKIR Questionnaire 8. QuickDASH stands for the short version of disabilities of the arm, shoulder and hand questionnaire. HQ-8 questions and total QuickDASH score evaluated preoperatively and postoperatively at three and 12 months (pooled). 0 is no problems and 100 is worst case scenario.
Values are median [IQR; 25^th^ – 75^th^ percentiles].

P-values based on Mann-Whitney U-test (age). Significant if p<0.05; marked as bold.

*^a^ Data missing in 1 patient in neurolysis group.*

*^b^ Data missing in 1 patient in nerve repair with sutures/nerve reconstruction/nerve transposition group
^c^ Data missing in 1 patient in nerve repair with sutures/nerve reconstruction/nerve transposition group.*

**Supplementary Table S4.** Pre- and postoperative response of HQ-8 questions and total QuickDASH score for patients surgically treated for neuroma in upper limb with postoperative response at three or 12 months pooled.

|  | **Preoperative (n=17)** | **Postoperative**  **(n=17)** | **P-value** |
| --- | --- | --- | --- |
| **Age (years)** | 52 [42-64] | 52 [42-64] | NA |
| **Pain on load** | 60 [40-80] | 50 [28-70] | 0.12 |
| **Pain on motion without load** | 35 [10-55] | 36 [5-60] | 0.57 |
| **Pain at rest** | 28 [2-50] | 31 [0-50] | 0.68 |
| **Stiffness ^a^** | 43 [15-63] | 47 [21-75] | 0.61 |
| **Weakness ^b^** | 50 [30-70] | 44 [10-70] | 0.48 |
| **Numbness/tingling in fingers ^c^** | 62 [42-90] | 38 [5-65] | **0.005** |
| **Cold Sensitivity ^d^** | 51 [10-85] | 44 [20-78] | 0.62 |
| **Ability to perform daily activities** | 50 [30-75] | 48 [25-65] | 0.45 |
| **Total QuickDASH score** | 48 [25-70] | 43 [26-64] | 0.11 |

HQ-8 questions are an abbreviation of HAKIR Questionnaire 8. QuickDASH stands for the short version of disabilities of the arm, shoulder and hand questionnaire. HQ-8 questions and total QuickDASH score evaluated preoperatively and postoperatively at three and 12 months (pooled). 0 is no problems and 100 is worst case scenario.
Values are median [IQR; 25^th^ – 75^th^ percentiles].

P-values based on paired samples with Wilcoxon rank sum test. Significant if p<0.05; marked as bold.

*^a^ Data missing in 1 patient in preoperative group.*

*^b^ Data missing in 1 patient in preoperative group.*

*^c^ Data missing in 1 patient in preoperative group.*

*^d^ Data missing in 1 patient in preoperative group.*

**Supplementary Table S5.** Postoperative response of HQ-8 questions and total QuickDASH score for patients surgically treated for neuroma in upper limb with data from three or 12 months pooled.

|  | **Neurolysis**  **(n=15)** | **Nerve repair with sutures/nerve reconstruction or**  **nerve transposition (n=12)** | **P-value** |
| --- | --- | --- | --- |
| **Age (years)** | 57 [37-61] | 51 [43-59] | 0.85 |
| **Sex (women/men)** | 10/5 (67/33) | 5/7 (42/58) | 0.35 |
| **Pain on load ^a^** | 68 [43-80] | 42 [33-70] | 0.31 |
| **Pain on motion without load ^b^** | 40 [10-63] | 25 [10-56] | 0.61 |
| **Pain at rest ^c^** | 37 [2-55] | 15 [0-41] | 0.28 |
| **Stiffness ^d^** | 62 [28-83] | 35 [6-75] | 0.19 |
| **Weakness ^d^** | 53 [13-83] | 25 [10-48] | 0.22 |
| **Numbness/tingling in fingers ^e^** | 55 [28-72] | 25 [0-58] | 0.16 |
| **Cold Sensitivity ^f^** | 70 [23-90] | 25 [1-58] | 0.12 |
| **Ability to perform daily activities ^g^** | 57 [11-80] | 35 [5-50] | **0.049** |
| **Total QuickDASH score ^h^** | 55 [26-66] | 30 [10-49] | 0.09 |

HQ-8 questions are an abbreviation of HAKIR Questionnaire 8. QuickDASH stands for the short version of disabilities of the arm, shoulder and hand questionnaire. HQ-8 questions and total QuickDASH score evaluated preoperatively and postoperatively at three and 12 months (pooled). 0 is no problems and 100 is worst case scenario.
Values are median [IQR; 25^th^ – 75^th^ percentiles].

P-values based on Mann-Whitney U-test (age). Significant if p<0.05; marked as bold.

*^a^ Data missing in 1 patient in nerve repair with sutures/nerve reconstruction/nerve transposition group.*

*^b^ Data missing in 1 patient in nerve repair with sutures/nerve reconstruction/nerve transposition group.*

*^c^ Data missing in 1 patient in nerve repair with sutures/nerve reconstruction/nerve transposition group.*

*^d^ Data missing in 1 patient in nerve repair with sutures/nerve reconstruction/nerve transposition group.*

*^e^ Data missing in 1 patient in nerve repair with sutures/nerve reconstruction/nerve transposition group.*

*^f^ Data missing in 2 patients in nerve repair with sutures/nerve reconstruction/nerve transposition group.*

*^g^ Data missing in 1 patient in nerve repair with sutures/nerve reconstruction/nerve transposition group.*

*^h^ Data missing in 1 patient in nerve repair with sutures/nerve reconstruction/nerve transposition group.*
